# Ocular Perfusion Pressure and the Risk of Open-Angle Glaucoma: Systematic Review and Meta-analysis

**DOI:** 10.1038/s41598-020-66914-w

**Published:** 2020-06-22

**Authors:** Ko Eun Kim, Sohee Oh, Sung Uk Baek, Seong Joon Ahn, Ki Ho Park, Jin Wook Jeoung

**Affiliations:** 1 Department of Ophthalmology, Nowon Eulji Medical Center, Eulji University, Seoul, Republic of Korea; 2 Medical Research Collaborating Center, Seoul Metropolitan Government-Seoul National University Boramae Medical Center, Seoul, Republic of Korea; 3 Department of Ophthalmology, Hallym University Sacred Heart Hospital, Hallym University College of Medicine, Anyang, Republic of Korea; 4 Department of Ophthalmology, Hanyang University Hospital, Hanyang University College of Medicine, Seoul, Republic of Korea; 5 Department of Ophthalmology, Seoul National University Hospital, Seoul National University College of Medicine, Seoul, Republic of Korea

**Keywords:** Eye diseases, Diseases, Medical research

## Abstract

Low ocular perfusion pressure (OPP) has been proposed as an important risk factor for glaucoma development and progression, but controversy still exists between studies. Therefore, we conducted a systematic review and meta-analysis to analyze the association between OPP and open-angle glaucoma (OAG). Studies were identified by searching PubMed and EMBASE databases. The pooled absolute and standardised mean difference in OPP between OAG patients and controls were evaluated using the random-effects model. Meta-regression analysis was conducted to investigate the factors associated with OPP difference between OAG patients and controls. A total of 43 studies were identified including 3,009 OAG patients, 369 patients with ocular hypertension, and 29,502 controls. The pooled absolute mean difference in OPP between OAG patients and controls was −2.52 mmHg (95% CI −4.06 to −0.98), meaning significantly lower OPP in OAG patients (*P* = 0.001). Subgroup analyses showed that OAG patients with baseline IOP > 21 mmHg (*P* = 0.019) and ocular hypertension patients also had significantly lower OPP than controls (*P* < 0.001), but such difference in OPP was not significant between OAG patients with baseline IOP of ≤21 mmHg and controls (*P* = 0.996). In conclusion, although no causal relationship was proven in the present study, our findings suggest that in patients with high baseline IOP, who already have a higher risk of glaucoma, low OPP might be another risk factor.

## Introduction

Glaucoma, the second worldwide leading cause of blindness^1^, is a progressive and chronic disease characterized by the degeneration of retinal ganglion cell and its axon with corresponding visual field defect^2^. Although therapeutic risk factors for preventing development and progression of glaucoma have been under wide investigation, lowering intraocular pressure (IOP) currently is the only effective treatment^3–5^.

Ocular perfusion pressure (OPP), the pressure to drive blood throughout the intraocular vasculature, with the degree of perfusion being influenced by the flow resistance, represents the blood flow and oxygen supplying the optic nerve head (ONH)^6,7^. Thus, it has long been proposed that a decrease in OPP may increase the vulnerability of optic disc, leading to an increased risk of glaucoma development or progression^8–12^. However, the association between OPP level and the risk of glaucoma has been debatable between studies. Some studies reported a significant association between low OPP and an increased risk of glaucoma^8–12^. In contrast, others have reported the statistically non-significant or limited impact of OPP on the risk of glaucoma^13,14^.

Another hindrance when referring to OPP in clinic is that as various levels of OPP have been reported depending on the study design, glaucoma type, and patient characteristics (e.g. presence of hypertension, use of anti-hypertensive medication), this has led to controversy over the significant difference in the level of OPP between glaucoma patients and controls. In light of these, we performed a systematic review and meta-analysis to investigate the pooled difference of OPP between glaucoma patients and controls and the association between mean OPP (MOPP) level and the risk of open-angle glaucoma (OAG).

## Materials and methods

### Search strategy and study selection

This study adhered to the PRISMA statement to follow the appropriate guidelines for systematic review and meta-analysis^15^. The Ovid interface was used to search for the keywords in the databases PubMed, EMBASE, and the Cochrane Library. The keywords for disease were “open-angle glaucoma”, “primary open-angle glaucoma”, “high tension glaucoma”, “normal tension glaucoma”, and “ocular hypertension. The keywords for ocular perfusion pressure were “ocular perfusion pressure”, “mean ocular perfusion pressure”, “systemic ocular perfusion pressure”, and “diastolic ocular perfusion pressure”. The following search terms were used: (glaucoma, open-angle [Medical Subject Headings {MeSH}] OR open angle glaucoma OR open-angle glaucoma OR OAG OR primary open angle glaucoma OR primary open-angle glaucoma OR POAG OR high tension glaucoma OR low tension glaucoma [MeSH] OR glaucoma, low tension OR low tension glaucoma OR normal tension glaucoma OR glaucoma, normal tension OR normal-tension glaucoma OR NTG OR ocular hypertension OR OHT) AND (ocular perfusion pressure OR OPP OR mean ocular perfusion pressure OR MOPP OR systolic ocular perfusion pressure OR SOPP OR diastolic ocular perfusion pressure OR DOPP). The literature search was conducted according to MeSH and no language restrictions were applied during the search. Two investigators (KEK, SJA) performed the literature search and study selection in an independent and masked fashion. Studies published before May 31 2019 were included. After screening titles and abstracts, full-text articles of eligible studies following the inclusion and exclusion criteria were attained.

Studies met the following criteria were included: (1) providing information on MOPP (2/3 [diastolic BP + 1/3 (systolic BP-diastolic BP)] – IOP) level represented as mean ± standard deviation (SD) in both controls and patients (2) IOP values represented as mean ± SD, measured by Goldmann applanation tonometry (3) open-angle glaucoma diagnosed with structural change (ONH, retinal nerve fiber layer) and corresponding functional changes, (4) ocular hypertension (OHT) diagnosed as IOP > 21 mmHg without any glaucomatous structural or functional damage. (5) studies approved by an institutional review board or ethics committee and followed the guidelines from the Declaration of Helsinki.

Exclusion criteria were: (1) studies reported an association between OPP and glaucoma in the form of correlation coefficients or odds ratio, (2) experimental studies involving non-human population, (3) angle-closure glaucoma or open-angle glaucoma with any secondary cause (e.g., uveitis, pseudoexfoliation syndrome), (4) glaucoma patients that underwent other ocular surgeries or received treatments other than topical IOP-lowering medication, (5) patients with uncontrolled hypertension, (6) papers not available in English, (7) studies involving patients less than 18 years of age, (8) abstracts or conference proceedings that were not published in peer-reviewed journals.

**Figure 1:**
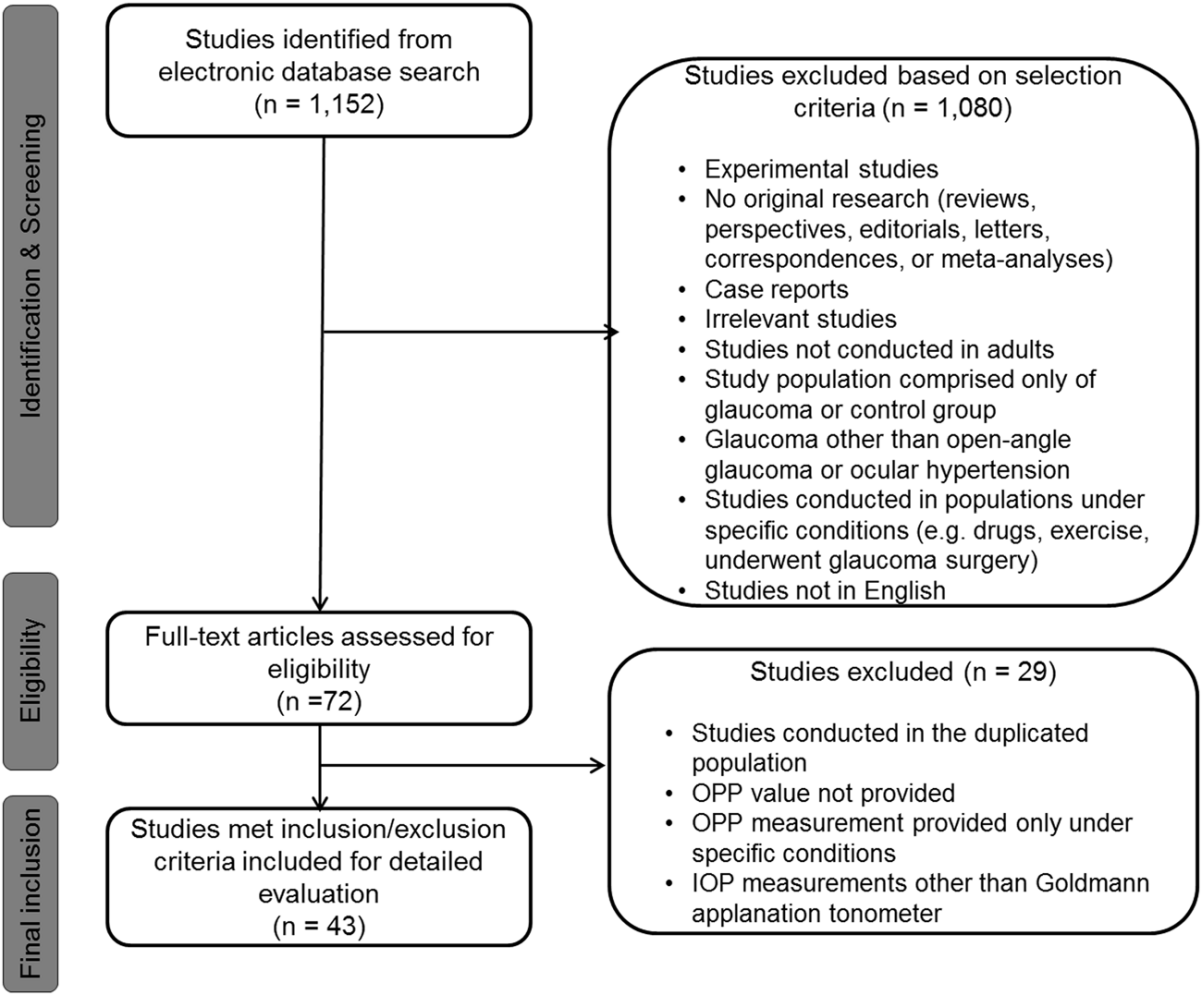
Flow diagram of the study selection process.

The study arms were divided into glaucoma/OHT and controls. The included studies had data on the following groups of glaucoma patients: (1) OAG (OAG without definite information on baseline IOP for inclusion criteria), (2) primary open-angle glaucoma (POAG; OAG patients with baseline IOP > 21 mmHg) and, (3) normal-tension glaucoma (NTG; OAG patients with baseline IOP ≤ 21 mmHg). The OAG arm in the present study combined OAG, POAG, and NTG patients. Additionally, we performed separate analyses for POAG, NTG, and OHT groups. Only controlled (stable) glaucoma patients were included. If more than one published article reported on the similar findings within the same study population by the same researcher group, the most recent publication or the publication with the higher level of evidence, or larger number of study subjects has been selected. If the IOP, BP, or OPP values were measured several times throughout the day, the first measurements were included in the analyses. The electronic search strategy and sequential exclusion are outlined in Fig. 1.

### Data extraction and quality assessment

Two investigators (KEK and SUB) independently extracted data in a masked manner using a data extraction form. Discrepancies between the investigators were resolved by the third investigator (JWJ). Following data were extracted from the studies: (1) study characteristics, including the year of publication, name of the first author, country, study design, number of included eyes, patient demographics; (2) type of glaucoma, including OAG, POAG, NTG, and OHT; (3) outcome measurements, including OPP (MOPP, systolic OPP, diastolic OPP), IOP, and BP (mean arterial pressure [MAP], systolic BP [SBP], diastolic BP [DBP]); (4) patient characteristics, including proportion of patients under IOP-lowering medication, proportion of patients with hypertension or under hypertension medication.

The quality of included studies was assessed using the Risk of Bias Assessment Tool for Nonrandomized Studies (RoBANS 2.0) method by two independent investigators (KEK and SUB)^16^. The RoBANS tool consists of eight domains: comparability of participants, selection of participants, confounding variables, intervention (exposure) measurement, blinding of outcome assessment, outcome evaluation, incomplete outcome data, and selective outcome reporting. The risk of bias for each domain was categorized as low risk, high risk, and unclear risk.

### Statistical methods

The pooled OPP difference, which is to say, the difference in OPP between the glaucoma and control groups from the meta-analysis of the included studies, was presented as the mean difference and the standardised mean difference with 95% confidence interval (CI). Heterogeneity in meta-analysis refers to the variation in study outcomes between or among studies. The I^2^ statistic, representing the percentage of variation across studies that is due to heterogeneity rather than to sampling error, was evaluated for the degree of unexplained variation in the OPP/glaucoma association^17,18^. The values can range from 0 to 100%, 0% indicating statistical homogeneity and 100% statistical heterogeneity. It has been suggested that the adjectives low, moderate, and high be ascribed to I^2^ values of 25, 50, and 75%, respectively^19^. Due to high levels of heterogeneity, differences in mean IOP, OPP, BP levels between glaucoma patients and control were analyzed using random-effects model, which assumes that the true underlying effect between studies varies^20^. Meta-regression analysis was used to find possible potential factors that could result in OPP difference between glaucoma patients and controls. Variables including age, gender (proportion of men), MAP, SBP, DBP and IOP were included in the analyses. For studies not having MAPs but only SBP and DBPs, MAPs were calculated as DBP + [1/3 (SBP – DBP)]. All statistical analyses used 95% CI and *P*-values with a cut-off point of 0.05. All statistical analyses were performed using the software package R version 3.6.2^21^.

## Results

### Characteristics of included studies

We identified 1,152 studies through database searches. After reviewing abstracts, we excluded 1,080 studies that were not relevant, leaving 72 studies for full-text evaluation (Fig. 1). Of these, 43 studies were finally included in the current systematic review and meta-analyses. Among them, 4 studies were population-based, cross-sectional studies^8,22–24^ and the others were hospital-based, clinical case-control studies^14,25–62^. These 43 studies included 3,009 patients with OAG (1,294 OAG, 926 POAG, and 789 NTG), 369 OHT patients, and 29,502 controls from 19 countries and their characteristics are summarized in the Table 1. The quality of the evidence was generally good, but the risk of bias caused by confounding variables was high in 12 (27.9%) studies, and unclear in 3 (7.0%) studies.

**Table 1:** Characteristics of subjects in included studies.

First author	Year	Hospital-based, clinical study	Glaucoma subtype/Control	No. of included eyes	MOPP (Mean ± SD)	IOP (Mean ± SD)	No. of subjects with HTN	No. of subjects on systemic HTN med
Mursch-Edlmayr AS	2019	Yes	NTG	9	53.6 ± 4.6	13.9 ± 1.6	—	—
			Control	9	54.3 ± 4.4	14.6 ± 2.4	—	—
Cantor E	2018	No (Population based, cross-sectional)	OAG	65	46.2 ± 10.1	15.8 ± 5.0	Included	Included
			Control	1,076	47.9 ± 7.8	14.3 ± 2.7	Included	Included
Tham YC	2018	No (Population based, cross-sectional)	OAG	293	55.9 ± 9.1	16.7 ± 4.7	152 HTN	—
			Control	19,294	55.8 ± 8.5	15.1 ± 3.2	6,114 HTN	—
Hidalgo-Aguirre M	2017	Yes	OAG	15	48.3 ± 5.8	15.5 ± 1.5	—	—
			OHT	6	44.1 ± 6.2	21.6 ± 4.8	—	—
			Control	10	48.3 ± 7.6	15.5 ± 1.5	—	—
Gao Y	2016	Yes	POAG	54	29.6 ± 4.5	28.3 ± 2.1	None	None
			NTG	67	43.5 ± 5.2	13.9 ± 1.6	None	None
			Control	54	44.7 ± 4.8	14.3 ± 1.9	None	None
Samsudin A	2016	Yes	NTG	31	60.5 ± 8.7	11.2 ± 2.6	17 HTN	—
			Control	15	62.9 ± 10.2	11.1 ± 2.1	None	None
Abegão Pinto L	2016	Yes	POAG	214	57.8 ± 10.7	14 ± 4.5	68 HTN	60 on med
			NTG	192	57.5 ± 11.9	11.8 ± 3.2	64 HTN	82 on med
			Control	140	53.1 ± 10.3	14.2 ± 3.9	—	—
Jonas JB	2015	No (Population based, cross-sectional)	OAG	119	48.8 ± 12	16.5 ± 5.8	—	—
			Control	4,425	46.9 ± 8.8	13.7 ± 3.2	—	—
Modrzejewska M	2015	Yes	POAG	56	40.62 ± 5.95	20.02 ± 4.11	—	—
			Control	54	55.11 ± 2.22	16.13 ± 1.25	—	—
Goharian I	2015	Yes	OAG	23	45.8 ± 5.8	14.4 ± 4.2	8 HTN	8 on med
			Control	22	45.8 ± 6.1	14.3 ± 3.3	7 HTN	7 on med
Abegão Pinto L	2014	Yes	POAG	74	54.4 ± 9.8	17.5 ± 4.2	—	—
			NTG	63	55.9 ± 10.3	15.6 ± 2.8	—	—
			Control	55	53.5 ± 9.6	17.1 ± 3.3	—	—
Sehi M	2014	Yes	OAG	30	46.1 ± 6.8	14.2 ± 3.9	9 HTN	9 on med
			Control	27	51.1 ± 6.7	13.9 ± 2.3	—	—
Willekens K	2014	Yes	POAG	88	57.9 ± 9.2	14.5 ± 4.3	—	—
			NTG	58	59.4 ± 8.5	11.9 ± 3	—	—
			Control	51	56.3 ± 7.7	13.6 ± 2.6	—	—
Abegão Pinto L	2013	Yes	POAG	86	57.4 ± 10	14.8 ± 5.0	—	—
			NTG	69	58.9 ± 9.3	12.3 ± 2.8	—	—
			Control	81	55.5 ± 9.9	16.0 ± 4.8	—	—
Figueiredo BP	2013	Yes	OAG	30	46.3 ± 7.9	19 ± 5.1	—	—
			OHT	30	41.5 ± 5.2	22.4 ± 2.1	—	—
			Control	30	50.2 ± 7	12.9 ± 2.2	—	—
Gugleta K	2013	Yes	POAG	50	51 ± 11	15.8 ± 4.6	Controlled HTN	—
			OHT	46	48 ± 10	21.5 ± 4	Controlled HTN	—
			Control	56	54 ± 10	13.5 ± 2.8	Controlled HTN	—
Gherghel D	2013	Yes	POAG	34	47.27 ± 7.48	25.44 ± 3.63	None	None
			NTG	30	48.8 ± 6.31	17.76 ± 2.56	None	None
			Control	53	50.27 ± 8.21	16.6 ± 3.34	None	None
Ramli N	2013	Yes	NTG	72	55.48 ± 6.84	14.87 ± 2.26	38 HTN	2 on beta-blocker
			Control	55	56.64 ± 5.60	14.57 ± 2.09	38 HTN	3 on beta-blocker
Wang J	2013	Yes	OAG	108	45.9 ± 7.9	15.0 ± 4.0 (median, IQR)	—	—
			OHT	45	42.6 ± 6.6	20.0 ± 4.0 (median, IQR)	—	—
			Control	56	45.3 ± 6.2	14.0 ± 4.1 (median, IQR)	—	—
Mroczkowska S	2013	Yes	POAG	19	41.87 ± 8.96	23.94 ± 2.00	—	—
			NTG	19	47.29 ± 8.82	17.40 ± 1.80	—	—
			Control	20	55.94 ± 13.98	15.05 ±2.48	—	-
Plange N	2012	Yes	POAG	27	47.5 ± 7.4	18.0 ± 3.0	—	—
			Control	15	48.3 ± 9.3	15.0 ± 2.0	—	—
Portmann N	2011	Yes	POAG	45	48 ± 11	17 ± 5	Exclude unstable HTN	Exclude unstable HTN
			OHT	45	49 ± 10	22 ± 4	Exclude unstable HTN	Exclude unstable HTN
			Control	45	54 ± 9	15 ± 2	Exclude unstable HTN	Exclude unstable HTN
Galassi F	2011	Yes	NTG	44	44.54 ± 2.81	17.79 ± 1.51	None	None
			Control	40	52.18 ±4.47	17.3 ± 1.09	None	None
Sehi M	2011	Yes	POAG	14	42 ± 7.1	23 ± 5.6	None	None
			Control	14	47.6 ± 6.1	15.4 ± 4.1	None	None
Garhöfer G	2010	Yes	POAG	252	66.0 ± 8.0	16.2 ± 2.1	—	—
			Control	198	68.0 ± 11.0	15.3 ± 2.1	—	—
Zheng Y	2010	No (Population based, cross-sectional)	OAG	131	51.6 ± 10.2	16.8 ± 5.9	94 HTN	32 on med
			Control	3,130	52.8 ± 9.3	15.3 ± 3.5	2,138 HTN	669 on med
Kim YK	2010	Yes	NTG	24	46.8 ± 5.6	13.4 ± 2.4	6 HTN	6 on med
			Control	22	49.2 ± 3.7	12.8 ± 3.1	None	None
Deokule S	2009	Yes	OAG	22	98.9 ± 11.6	14 ± 5.1	—	—
			Control	21	100.5 ± 21.3	12.7 ± 4.7	—	—
Pemp B	2009	Yes	POAG	15	47.9 ± 7.5	16.7 ± 2.1	Controlled HTN	1 beta blocker
			Control	15	51.9 ± 7.9	15.8 ± 2.5	Controlled HTN	1 beta blocker
Resch H	2009	Yes	POAG	14	42 ± 8	17 ± 3	—	—
			Control	14	47 ± 4	14 ± 3	None	None
Plange N	2008	Yes	NTG	35	48 ± 10	16 ± 3	—	—
			Control	35	47 ± 7	16 ± 2	—	—
Januleviciene I	2008	Yes	POAG	60	54.2 ± 8.2	21.28 ± 3.1	—	—
			Control	30	59.1 ± 9.6	15.47 ± 1.9	—	—
Galassi F	2008	Yes	POAG	41	82.5 ± 7.31	14.49 ± 2.96	None	None
			Control	38	81.64 ±6.12	14.32 ± 2.05	None	None
Feke GT	2008	Yes	OAG	18	51.4 ± 7.9	14 ± 3	—	3 on med
			Control	8	46.4 ± 6.0	13 ± 3	None	None
Riva CE	2004	Yes	OAG	13	45.00 ± 6.00	19.00 ± 3.00	—	—
			OHT	29	47.00 ± 5.00	18.00 ± 2.00	—	—
			Control	16	48.00 ± 6.00	16.00 ± 2.00	—	—
Gherghel D	2004	Yes	POAG	24	39.63 ± 8.56	23.63 ± 4.89	—	—
			Control	22	44.30 ± 9.92	17.95 ± 3.74	None	None
Galassi F	2004	Yes	POAG	38	51.21 ± 5.66	16.6 ± 5.1	None	None
			Control	46	53.26 ± 6.40	14.1 ± 2.8	None	None
Fuchsjäger-Mayrl G	2004	Yes	OAG	49	39.0 ± 7.2	22.6 ± 2.9	—	—
			OHT	91	40.6 ± 9	23.2 ± 2.8	—	—
			Control	102	51.8 ± 6.3	14.5 ± 2.2	—	—
Hosking SL	2004	Yes	POAG1	12	50.8 ± 14.0	15.4 ± 4.1	None	None
			POAG2	13	48.9 ± 5.7	14.8 ± 3.5	None	None
			Control1	16	47.5 ± 4.9	14.4 ± 2.5	None	None
			Control2	15	48.0 ± 5.4	15.1 ± 2.5	None	None
Okuno T	2004	Yes	NTG	12	52 ± 3	14.1 ± 0.7 (morning, mean ± SE)	None	None
			Control	12	50 ± 3	14.8 ± 1.0 (morning, mean ± SE)	None	None
Kerr J	2003	Yes	POAG	24	46.4 ± 13.1	28.6 ± 4.2	2 HTN	None
			OHT (high risk)	23	47.0 ± 13.5	28.3 ± 3.1	5 HTN	None
			OHT (low risk)	22	53.3 ± 8.5	22.1 ± 1.4	5 HTN	None
			Control	23	59.1 ± 10.8	16.0 ± 2.3	2 HTN	None
Hafez AS	2003	Yes	OAG	20	43.2 ± 6.1	22.2 ± 4.2	6 HTN	6 on med
			OHT	20	42.8 ± 10.6	28.7 ± 3.9	4 HTN	4 on med
			Control	20	48.2 ± 7.2	16.9 ± 2.6	2 HTN	2 on med
Duijm HF	1997	Yes	POAG	48	77.7 ± 17.9	30.4 ± 11.3	—	—
			NTG	46	86.2 ± 11.9	18.1 ± 2.7	—	—
			OHT	12	76.8 ± 15.4	26.5 ± 6.1	—	—
			Control	22	83.4 ± 9.1	13.7 ± 2.3	—	—

MOPP = mean ocular perfusion pressure; IOP = intraocular pressure; SD = standard deviation; OAG = open-angle glaucoma; POAG = primary open-angle glaucoma (OAG with baseline IOP of >21 mmHg); NTG = normal-tension glaucoma (OAG with baseline IOP of ≤21 mmHg); OHT = ocular hypertension; HTN = hypertension; med = medication; SE = standard error; IQR: inter-quartile range.

### Ocular perfusion pressure difference between glaucoma patients and control groups

**Figure 2:**
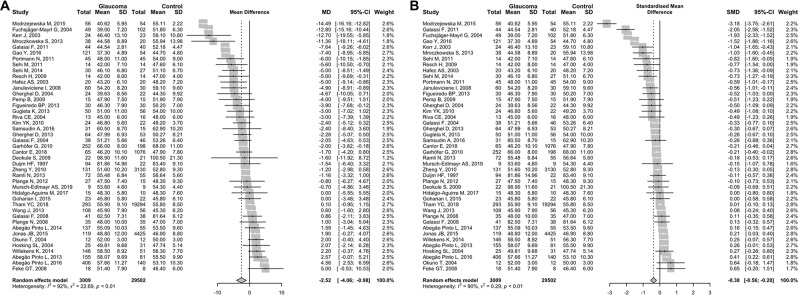
Random-effects meta-analysis of ocular perfusion pressure (OPP) difference between open-angle glaucoma patients and controls. Pooled OPP difference was presented as (**A**) the mean difference (MD) and (**B**) the standardised mean difference (SMD) with 95% confidence interval (CI). SD = standard deviation.

The main outcome of the present study was the difference in OPP (measured in mmHg) between patients with and without OAG and its significance. The pooled average difference in OPP between patients with and without OAG was −2.52 mmHg (95% CI, −4.06 to −0.98, *P* = 0.001), with a high degree of heterogeneity (I^2^ = 92.3%), presented in Fig. 2A. The pooled standardised average difference in OPP between OAG patients and control was −0.38 (95% CI, −0.56 to −0.20, *P* < 0.001), also with high degree of heterogeneity (I^2^ = 90.1%, Fig. 2B). These showed that OAG patients had significantly lower OPP than controls.

**Figure 3:**
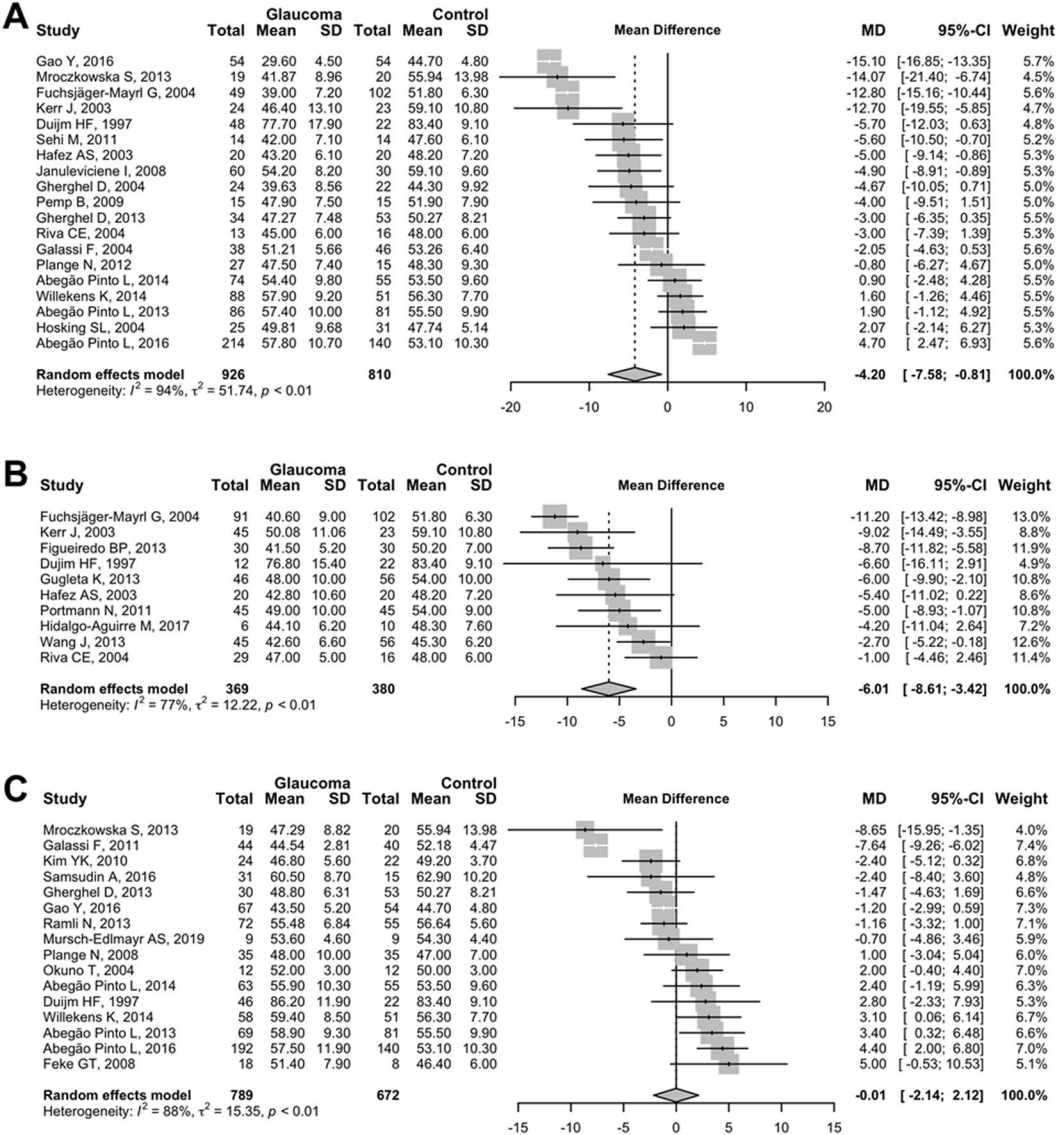
Random-effects meta-analysis of the mean ocular perfusion pressure (OPP) difference between (**A**) primary open-angle glaucoma (open-angle glaucoma [OAG] patients with baseline intraocular pressure [IOP] of>21 mmHg), (**B**) ocular hypertension, (**C**) normal-tension glaucoma (OAG patients with baseline IOP of ≤ 21 mmHg) and controls. SD = standard deviation; MD = mean difference; CI = confidence interval.

**Figure 4:**
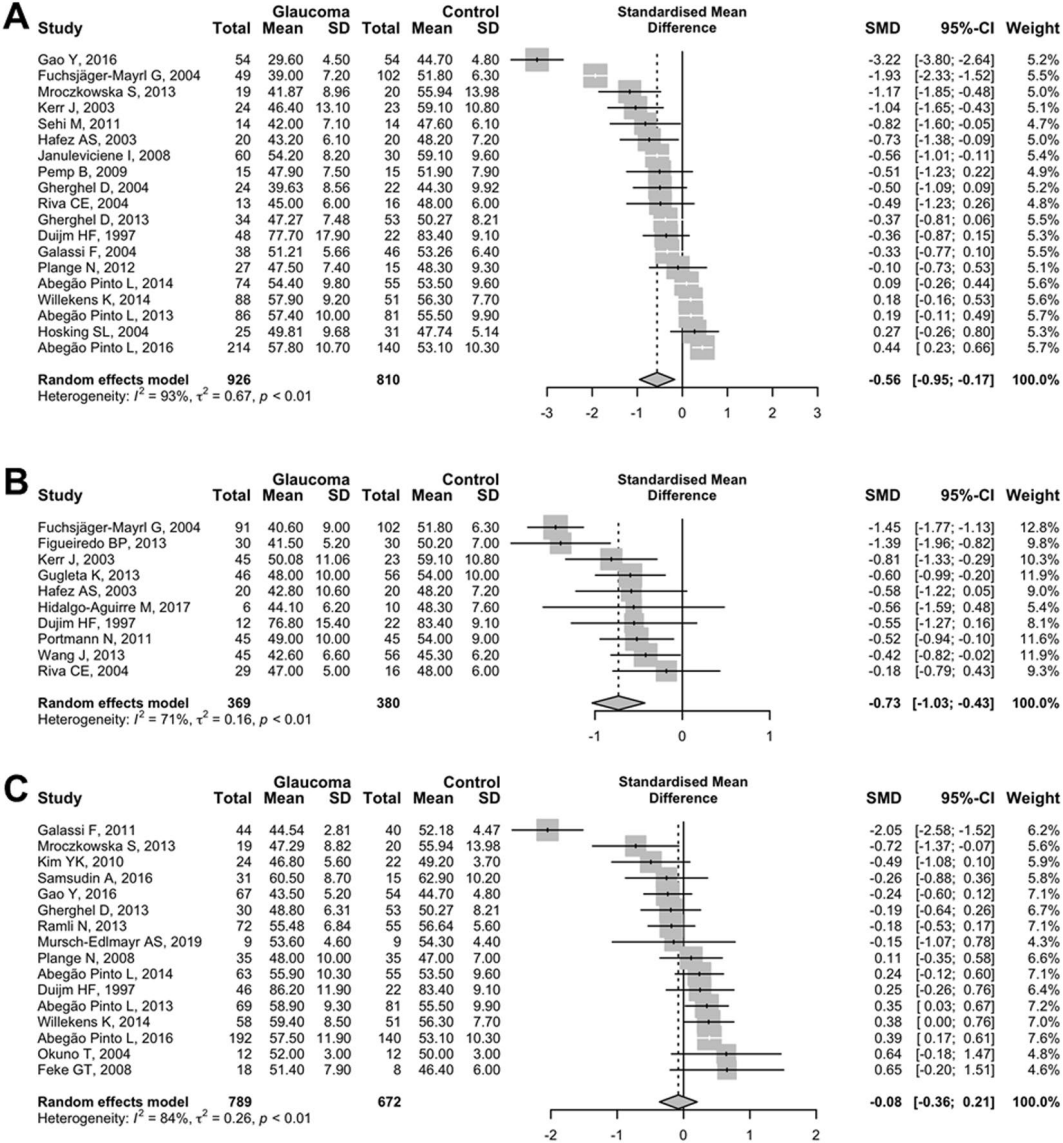
Random-effects meta-analysis of the standardised mean ocular perfusion pressure (OPP) difference between (**A**) primary open-angle glaucoma (open-angle glaucoma [OAG] patients with baseline intraocular pressure [IOP] of>21 mmHg), (**B**) ocular hypertension, (**C**) normal-tension glaucoma (OAG patients with baseline IOP of ≤21 mmHg) and controls. SD = standard deviation; SMD = standardised mean difference; CI = confidence interval.

Subgroup analyses showed that POAG patients had significantly lower pooled average OPP compared to controls (−4.20 mmHg, 95% CI −7.58 to −0.81, *P* = 0.019, Fig. 3A) and the similar trend was found in patients with OHT (−6.01 mmHg, 95% CI −8.61 to −3.42, *P* < 0.001, Fig. 3B). However, the pooled average difference in OPP between NTG patients and controls was not significant (−0.01 mmHg, 95% CI −2.14 to 2.12 mmHg, *P* = 0.996, Fig. 3C). The standardised mean difference in OPP also showed similar relationships between the subgroup of glaucoma patients and controls (Fig. 4A–C).

### Additional analyses

**Figure 5:**
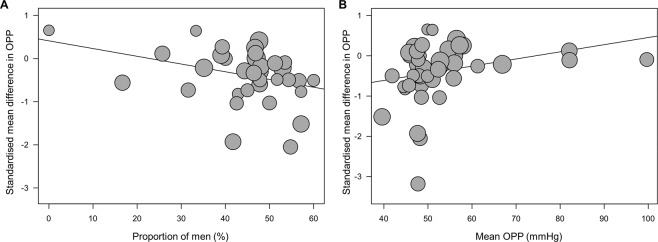
Random-effects meta-regression of standardised mean difference in ocular perfusion pressure (OPP) between patients with open-angle glaucoma (OAG) and controls according to (**A**) proportion of men and (**B**) mean OPP level. The line represents a line of best fit from meta-regression analysis. This suggests that the standardised mean difference in OPP levels between patients with OAG and controls was the largest in study population with large proportion of men (*P* = 0.040) and low mean OPP level (*P* = 0.029).

The study that contributed the most to the heterogeneity for the OPP difference between controls and OAG patients was Mroczkowska *et al*.^36^ Meta-regression analyses using random-effects model were performed to investigate the potential risk factors associated with the pooled standardised average difference in OPP between OAG patients and controls. Random effects meta-regression analyses showed that age, systolic BP, diastolic BP, mean arterial pressure, and study design were not significantly associated with the standardised mean difference in OPP. However, studies with larger proportion of men showed increasing trend of standardised mean difference in OPP (*P* = 0.040, Fig. 5A) and the OPP difference was larger in studies with lower mean OPP level (*P* = 0.029, Fig. 5B).

## Discussion

Our systematic review and meta-analysis showed that patients with OAG had lower mean OPP compared to controls. The pooled mean absolute difference in OPP level between OAG patients and controls was −2.52 mmHg. Moreover, patients with POAG and OHT also showed significantly lower OPP than controls, with the pooled mean absolute difference of −4.20 mmHg and −6.01 mmHg, respectively. However, this trend of the relationship was not significant in NTG patients. We concluded that low OPP may be a significant risk factor for OAG patients with high baseline IOP, and thus, control of IOP leading to appropriate OPP may be important in terms of regulating vascular factors for glaucoma treatment.

Vascular factors have long been suspected of playing an important role in the glaucomatous process in addition to IOP. In this aspect, previous studies have reported a significant association between low OPP and glaucoma. Low OPP represents hypo-perfusion to ONH, ultimately leading to ONH deprived of nutrition and oxygenation. However, several studies have shown no association between them^14^. This may be due to the fact that IOP and BP, the major constitutes of OPP, were measured in different clinical settings (e.g. study population, types of glaucoma, use of topical drugs). Moreover, various BP- and OPP-related parameters (e.g., MOPP, systolic OPP, diastolic OPP, MAP, SBP, DBP) were used, which rendered interpretation of the effect of OPP on glaucoma more complex, in line with the study by Barbosa-Breda *et al*.^63^ Therefore, to address the gap in consideration of heterogeneity between studies, we conducted systematic review and meta-analysis and confirmed that OAG patients had significantly lower MOPP than controls.

Our results showed that POAG patients, whose baseline IOP of more than 21 mmHg, showed significantly lower OPP than controls. Previous studies have suggested that NTG patients may be more affected by ischemic injury associated with vascular factors than mechanical injury by elevated IOP^44,64–69^. In this aspect, one may expect significantly lower OPP in NTG patients than in controls. However, this was not proven in the present study. Following reasons may explain our findings. First, OPP itself is not the only vascular factor causing ischemic injury to the optic nerve head. Moreover, several studies reported that OPP fluctuation or degree of its variability, rather than one single OPP value may be more important in development and progression of NTG^65,70,71^. Second, several studies have suggested that NTG patients may have vascular dysregulation or weak vascular regulating system to defense against provocative stimulation. Thus, their vulnerability to vascular insults may not be revealed under normal (resting) condition^14,72^. All of the included studies measured OPP at resting and sitting position, and thus, our results could not reveal the OPP results in NTG patients under provocative stimulation. Third, the present study only included studies with definite MOPP values presented as mean ± SD. Thus, several large, population-based studies including Baltimore Eye Survey^73^, Egna-Neumarkt Study^12^, Los Angeles Latino Eye Study^74^ which reported the association between low OPP and increased risk of glaucoma in terms of odds ratio were excluded. Since these studies also had a large proportion of patients with baseline IOP less than 21 mmHg, these could have affected the present meta-analysis.

The actual OPP should be determined by the difference between arterial pressure at the entrance to the eye and the venous pressure at the exit of the eye. However, currently available methods cannot directly measure such pressures. Therefore, OPP has been estimated by the difference between arterial pressure measured in the arm and IOP, which may not reflect actual measures. Based on current equation, either decrease in BP or increase in IOP may influence the decrease in OPP. However, these parameters cannot be evaluated separately for the association with glaucoma, since they are all included in the same equation. We conducted direct comparison of OPP, IOP, and BP between POAG and NTG subgroups in an attempt to explain the effects of these parameters on OPP difference. However, it was not possible, since only 18.6% (8/43) of studies had both POAG and NTG groups. Also, some studies presented “untreated” IOP values, while others presented “treated” IOP values for the subjects’ baseline characteristics. Thus, we indirectly calculated the pooled average OPP, IOP, and BP differences between POAG and NTG groups using network meta-analysis (Supplemental Table S1)^75^. Despite the high heterogeneity and the limited number of studies, we confirmed that the POAG group showed lower OPP and higher IOP compared with the NTG group. By contrast, there was no significant difference in pooled average BP difference between POAG and NTG patients by network meta-analysis. These additional findings seem to imply that high IOP might be the basis for low OPP in patients with POAG.

Several studies reported that diastolic BP is more important in determining OPP than other BP parameters. As the degree of BP is larger than that of IOP, OPP may be more sensitive to changes in BP than those in IOP. We used meta-regression analyses to examine the potential evidence as to which BP parameter would be associated with OPP difference. However, none of the BP parameters showed any association with OPP difference. This could be attributable to the fact that all of the studies included patients with no hypertension or hypertension under controlled BP with or without medication. Another possible cause is the fact that not all of the studies had available BP values. To overcome the limitation that only 32.6% (14/43) studies had mean arterial pressures, we even calculated them additionally based on systolic BP and diastolic BP provided in 65.1% (28/43) of studies, but the association was insignificant. As only mean OPP was used in the analyses, the future investigation is needed on whether BP parameters could have effect on other OPP parameters (e.g. diastolic, systolic).

Several limitations should be considered for the interpretation of our results. First, our meta-analyses are based on cross-sectional studies. Thus, despite a significant association between low OPP and glaucoma, our results cannot provide evidence for a causal relationship between them. The Barbados Eye Study, a prospective, longitudinal study reported that low MOPP, systolic OPP, and diastolic OPP were all associated with a higher risk of developing glaucoma at 4 and 9 years of follow-up^10^. Despite this, further longitudinal studies providing sufficient clinical evidence are needed to address this causal relationship. Second, the heterogeneity of pooled studies was substantial. This may result from differences in study population, study design, and participant characteristics. We used random-effects analyses to overcome such limitation and no publication bias was detected by Egger’s test. Finally, the present study showed the absolute mean difference of OPP as approximately 2 mmHg between OAG patients and controls, but these were based on single measurements. Thus, further investigation is required to prove the clinical relevance of OPP fluctuation or dynamic range of OPP on glaucoma.

In conclusion, our systematic review and meta-analysis point to evidence for low OPP in OAG patients, which is in line with the current notion that decreased vascular supply to the ONH may increase its vulnerability to glaucomatous structural damage. Additionally, among OAG patients, those with high baseline IOP particularly, rather than those with low baseline IOP, showed significantly lower OPP than the controls. Although further investigation might be needed, our results imply that in patients with high baseline IOP, who already have a higher risk of glaucoma, low OPP might be another risk factor.

## Supplementary information

Supplementary information.
