# Supplementary material for: Ocular Perfusion Pressure and the Risk of Open-Angle Glaucoma: Systematic Review and Meta-analysis
Source: Sci Rep. 2020 Jun 22;10:10056. doi: 10.1038/s41598-020-66914-w (PMC7308312; doi:10.1038/s41598-020-66914-w)
Supplement: Supplementary file 1 — Supplementary information. [file 41598_2020_66914_MOESM1_ESM.pdf]

Supplementary Information for

Ocular Perfusion Pressure and the Risk of Open-Angle Glaucoma: Systematic Review and Meta-analysis

Ko Eun Kim[1], Sohee Oh[2], Sung Uk Baek[3], Seong Joon Ahn[4], Ki Ho Park[5], Jin Wook Jeoung[5]

[1]Department of Ophthalmology, Nowon Eulji Medical Center, Eulji University, Seoul, Republic of Korea

[2]Department of Biostatistics, Seoul National University Boramae Hospital, Seoul, Republic of Korea

[3]Department of Ophthalmology, Hallym University Sacred Heart Hospital, Hallym University College of Medicine, Anyang, Korea.

[4]Department of Ophthalmology, Hanyang University Hospital, Hanyang University College of Medicine, Seoul, Republic of Korea

[5]Department of Ophthalmology, Seoul National University Hospital, Seoul National University College of Medicine, Seoul, Republic of Korea

Supplemental Table S1. Pooled mean ocular perfusion pressure (OPP), intraocular pressure (IOP), and blood pressure (BP) difference between primary open-angle glaucoma (POAG; OAG patients with baseline IOP >21 mmHg) and normal-tension glaucoma (NTG; OAG patients with baseline IOP ≤21 mmHg) groups.

POAG vs. NTG	Number of studies	Pooled mean difference (POAG-NTG; mmHg)	Pooled standardized mean difference (POAG-NTG; mmHg)
**OPP**			
All studies	35	-4.11 (-8.12 – -0.10)<br>***P* = 0.045**, I^2 = 92.6%	-0.47 (-0.97 – 0.02)<br>*P* = 0.061, I^2 = 90.5%
**IOP**			
Studies provided with untreated IOP	18	8.34 (5.92 – 10.75)<br>***P* <0.001**, I^2 = 95.2%	2.54 (1.59 – 3.49)<br>***P* <0.001**, I^2 = 91.2%
Studies provided with treated IOP	17	2.83 (1.31 – 4.36)<br>***P* <0.001**, I^2 = 83.1%	0.84 (0.41 – 1.26)<br>***P* <0.001**, I^2 = 77.3%
**BP**			
Studies provided with systolic BP	26	1.62 (-1.90 – 5.14)<br>*P* = 0.367, I^2 = 43.1%	0.09 (-0.12 – 0.30)<br>*P* = 0.418, I^2 = 37.1%
Studies provided with diastolic BP	26	1.40 (-1.34 – 4.14)<br>*P* = 0.316, I^2 = 61.2%	0.14 (-0.10 – 0.38)<br>*P* = 0.258, I^2 = 51.7%
Studies provided with mean arterial pressure	7	0.43 (-4.71 – 5.57)<br>*P* = 0.869, I^2 = 2.0%	-0.04 (-0.52 – 0.44)<br>*P* = 0.876, I^2= 0.0%

95% confidence interval is in parentheses.

Statistical significance of *P* < 0.05 is indicated in bold.
